# Bouncing back from stress: objective markers of expressive flexibility and resilience in emergency healthcare workers using computer vision

**DOI:** 10.1038/s44277-026-00067-y

**Published:** 2026-07-15

**Authors:** Charlotte E. Hilberdink, Yiwen Zhao, Scott McKernan, Sapir Gershov, Victoria Mueller, Stephen P. Wall, Katharina Schultebraucks

**Affiliations:** 1 Department of Psychiatry, New York University Grossman School of Medicine, New York, NY USA; 2 Department of Psychology, The New School for Social Research, New York, NY USA; 3 Department of Emergency Medicine, New York University Grossman School of Medicine, New York, NY USA; 4 Division of Healthcare Delivery Science, Department of Population Health, New York University Grossman School of Medicine, New York, NY USA

**Keywords:** Human behaviour, Risk factors, Trauma

## Abstract

Healthcare workers (HCWs) in emergency departments face significant mental health risk due to chronic stressors and repeated trauma, yet symptom underreporting and bias in self-reports hinder accurate assessments. Expressive flexibility, the ability to dynamically modulate and recover from stressor-related changes in emotional arousal as reflected in observable behavior, has been linked to resilience. This NIH-funded study (R01HL156134) utilized digital phenotyping and computer vision to analyze dynamic facial expressivity during video-recorded interviews about work-related stressful situations with 240 HCWs (278 assessments). Participants additionally completed validated questionnaires to assess burnout, PTSD, depression, anxiety, and resilience. Latent profile analysis revealed two clinical phenotypes: At-risk (57.6%) and Resilient/Adaptive (42.4%). Machine learning models demonstrated high classification performance (accuracy = 0.83 ± 0.06, F1-score = 0.87 ± 0.05). Our findings indicate that digital biomarkers of temporal facial dynamics may serve as objective behavioral proxies of expressive flexibility, potentially capturing dynamics consistent with underlying stress-regulatory processes. These findings highlight their potential to improve identification of resilience-related phenotypes and support well-being and mental health in HCWs.

## Lay Summary

Emergency department healthcare workers face high levels of stress and trauma, but mental health difficulties are often underreported. Using computer vision to analyze facial expressions during interviews, we identified dynamic expressivity patterns linked to resilience and mental health risk. We were able to accurately distinguish resilient from at-risk workers, suggesting that digital biomarkers of facial expressions may offer a scalable, non-invasive way to improve early identification of mental health needs and support well-being in high-stress professions.

## Introduction

Emergency department healthcare workers (ED HCWs) are constantly exposed to many stressors while operating under rapidly changing and demanding conditions. The cumulative toll of chronic risks (e.g., persistent high workload, patient violence, secondary trauma exposure, ethical dilemmas, and emotional distress of patients and families) contributes to substantial psychological hardship among ED HCWs worldwide [1–4]. Although many HCWs in emergency medicine show remarkable resilience and adaptability and continue to deliver the highest-quality healthcare [5–7], their true psychological symptom rates may be underestimated as they are often underreported [8–10]. Given the central role resilience plays in sustaining well-being and high performance in challenging environments [11], fostering resilience in ED HCWs is a critical priority.

Resilience (i.e., the capacity to flexibly ‘bounce back’ from stressful experiences) depends on the effective and adaptive use of emotion regulation strategies [12, 13] and is considered a core mechanism of coping [14]. Rather than reflecting a static trait, resilience can be understood as the dynamic ability to adjust emotional responses to situational demands [15–17]. For ED HCWs, resilience seems to be sustained by their ability to continuously adjust their emotional responses while maintaining a professional demeanor in high-stakes environments while sustaining interpersonal relationships during critical encounters with patients and caregivers [18–20]. Indeed, flexible regulation of emotions in HCWs serves as a protective factor to withstand job-related stress and buffer against negative mental health outcomes, promoting psychological well-being [6, 21, 22]. Particularly, expressive flexibility, which includes the ability to both enhance and suppress facial expressions depending on contextual demands [23, 24], is a central behavioral component to adapt responses to emotional arousal for resilient outcomes [25, 26]. During stressful situations, individuals experience fluctuations in emotional arousal, and resilience depends partly on how flexibly they can modulate and recover from these changes [14, 27]. Expressive flexibility reflects this capacity in observable behavior, capturing how individuals dynamically adjust their emotional responses in real time and “bounce back” following stressors [25, 28]. Importantly, expressive behavior offers a measurable window into these adaptive processes without requiring direct assessment of underlying biological systems [29, 30]. Facial expressions dynamically track changes in emotional arousal and affective states [31], and the ability to flexibly modulate these expressions may reflect resilience-related regulatory capacity [32].

Previous research in trauma-exposed individuals has shown that successful adaptive functioning is related to expressive flexibility. For example, a positive relationship was found between expressive flexibility and long-term adjustment, and an inverse association with psychological distress [28]. Similarly, a study in HCWs found that expressive flexibility was protective against depressive, stress, and anxious symptoms during the COVID-19 pandemic [33], and associated with reduced vulnerability to PTSD among first responders following duty-related trauma exposure [34]. On the other hand, rigidity or diminished expressive flexibility was associated with greater severity of PTSD and depressive symptoms [35, 36]. However, these findings relied solely on self-reports of expressive flexibility, which may be prone to similar reporting biases that affect and challenge self-reported mental health assessments [10, 37]. Such biases likely reflect stigma and professional norms that valorize stoicism in high-performing medical environments. These tendencies towards underreporting may, in turn, delay diagnosis, reduce help-seeking, and hinder effective and timely treatment [38–40], and could ultimately result in compromised healthcare quality and patient safety [41, 42].

Digital phenotyping from video data that reflect quantified facial expressivity patterns could therefore offer promising unobtrusive and non-invasive proxies for expressive flexibility and resilience [26, 43]. Digital biomarkers derived from facial dynamics could reflect behavioral proxies of resilience-related regulatory processes, quantifying how individuals respond to and recover from stressors in ecologically valid contexts. Capturing facial expressive flexibility from passively observed data may offer a complementary approach to help improve identification of ED HCWs in need of support for underrecognized mental health challenges. Additionally, it might lessen the pressure and discomfort of self-reported mental health disclosures in high-stigma contexts, particularly when tied to support systems that prioritize and ensure privacy that better address their needs.

In this study, we utilized advanced computer vision to assess facial expressive flexibility during a video interview about stressful work events. We moved beyond traditional methods that average facial expressions at a set time period or timepoint by quantifying the temporal dynamics of facial expressivity (i.e., variability, state transitions, and time-resolved changes in affective signals), offering crucial insight into the nuanced ‘bounce back’ from emotional provocation with clinical and predictive value. We hypothesized that ED HCWs with greater expressive flexibility (i.e., more dynamic facial expressivity) would show higher resilience and fewer psychological symptoms; flexibility rather than ‘static’ expressivity was expected to identify behavioral markers of adaptive emotional responding and hold promise as a measurable indicator of resilience-related processes in clinical practice. This approach could offer novel insights into HCWs’ psychological resilience and may advance digital phenotyping for mental health monitoring, early detection, and prevention in high-stress professions.

## Materials & methods

### Participants

Participants in this study were HCWs who work in the ED and Trauma Unit in and around New York City (NYC) and were enrolled to an ongoing NIH-funded longitudinal observation study (Early Signs, R01HL156134), between April 2022 until April 2025. The primary aim of the Early Signs study is to examine burnout and cognitive functioning in ED HCWs. Participants were eligible for enrollment if they 1) were 18 years and older; 2) worked full-time clinical hours (including temporarily part-time work due to temporary leave or burnout); 3) worked directly with patients; 4) were fluent in English. Participants were excluded if they were a medical student. ED HCWs were recruited through in-person research staff visits to EDs across academic and public hospital systems in and around the NYC area, and through online advertisement. All participants signed informed consent forms and were reimbursed for their participation. The Institutional Review Board of NYU Grossman School of Medicine approved the study.

### Procedure

A total of N = 249 participants completed 288 in-person assessments during which current mental health status was evaluated using several self-report questionnaires (for data availability flowchart, see Supplementary Material S1). Also, a 15 min video-recorded semi-structured interview about stressful work experiences and medical decision making was conducted using a HIPAA-compliant video platform (WEBEX). A research coordinator conducted the interview virtually with the participant alone in a separate room. The interview included five questions designed to elicit recall of emotionally salient and potentially stressful experiences: 1) “Tell me about a case you were involved with in the ED where you felt there was an unexpected and bad patient outcome?”; 2) “Tell me how the patient outcome made you feel as the responsible provider?”; 3) “Has this patient case changed your view of yourself as a clinician and how you approach patient care?”; 4) “Tell me about a situation where you asked yourself, am I given enough recognition for my efforts?”; and 5) “What are your expectations about your role and your work as an ED clinician in the future?”. Participants had approximately three minutes response time per question and were instructed to answer the question until they were interrupted with the next question. Additionally, participants were instructed to consider the question in terms of their role in patient care in case the wording did not seem to directly apply to them. If participants had difficulty answering the question, they were prompted with an open-ended follow-up question to elaborate or share other relevant situations related to the question.

### Measures

Resilient coping was assessed using the Brief Resilient Coping Scale (BRCS sum score [44]). Burnout symptoms were measured using the abbreviated Maslach Burnout Inventory (MBI-9 [45]) for Personal Achievement (PA subdomain score), Emotional Exhaustion (EE subdomain score), and Depersonalization (DEP subdomain score). Depressive symptoms in the past 2 weeks were assessed using the Patient Health Questionnaire (PHQ-8 sum score [46]). Anxiety symptoms in the past 2 weeks were assessed using the Generalized Anxiety Disorder 7-item scale (GAD-7 sum score [47]). PTSD symptoms in the past month, related to COVID-19 or another traumatic work-related experience, were measured using the PTSD Checklist for DSM-5 (PCL-5 sum score [48]). Higher scores indicated higher symptom levels, although for PA lower subdomain scores indicated higher burnout levels.

### Statistical approach

#### Latent profile analysis (LPA)

To identify comprehensive latent profiles of subgroups who exhibit similar symptom patterns based on their occurrence and severity levels [49], we performed latent profile analysis (LPA) in Python (v3.12.4, ‘scikit-learn’ library v1.4.2). Features included in the LPA were standardized BRCS, PHQ-8, GAD-7, and PCL-5 sum scores and MBI-9 PA, EE, and DEP subdomain scores. Data were excluded in case of missing mental health questionnaire data (7 assessments from n = 7 participants) and changed eligibility after enrollment (3 assessments from n = 2 participants), resulting in 278 assessments from N = 240 participants for the LPA analysis. Additionally, features were checked for near-zero variance (threshold=0.1) prior to analysis. Model selection was based on comparisons across multiple class solutions using standard fit indices and entropy. Information on best-fitting model evaluation is presented in Supplementary Material S2. A sensitivity analysis was performed, excluding multivariate outliers (11 assessments from n = 10 participants) based on the Mahalanobis Distance (threshold=0.01; see Supplementary Material S3).

Differences in continuous data for mental health status between final profiles were assessed using Linear Mixed Models (LMM) with Restricted Maximum Likelihood (REML) in SPSS (v28.0.1.1, IBM SPSS Statistics Software). Best-fitted model with First-Order Autoregressive covariance structure had lowest values for -2 Log Likelihood, AIC and BIC criteria compared to models with Unstructured or Diagonal covariance structure.

#### Temporal feature modeling

##### Video-recording preprocessing

After removing unusable video data, due to absence of a visible face in 3 videos from n = 3 participants and incomplete interview data for 107 videos from n = 107 participants, a total of 168 video recordings of completed interviews from a subgroup of N = 133 participants were processed (Table 1), and each interview was addressed as separate observation. Each recording was divided into 5 segments, corresponding to each interview question.

**Table 1:** Demographic characteristics for the entire latent profile sample and for the sample used for the prediction analysis.

	Latent profile sample		Temporal Feature Modeling sample
**Participants**	240		133
**Age** (years)	37.2 (10.8)^g^		33.5 (8.0)^g^
**Sex at birth**			
Female	159 (66.3%)		81 (60.9%)
Male	81 (33.8%)		52 (39.1%)
**Race**			
White	105 (43.8%)		60 (45.1%)
Black or African American	39 (16.3%)		22 (16.5%)
Asian	55 (22.9%)		33 (24.8%)
Middle Eastern or North African	3 (1.3%)		1 (0.8%)
American Indian/Native American	2 (0.8%)		1 (0.8%)
Multiracial	11 (4.6%)		7 (5.3%)
Other	9 (3.8%)		2 (1.5%)
Unknown	4 (1.7%)		1 (0.8%)
Declined to respond	12 (5.0%)		6 (4.5%)
**Ethnicity**			
Hispanic or Latino	41 (17.1%)		17 (12.8%)
Non-Hispanic or -Latino	193 (80.4%)		111 (83.5%)
Declined to respond	6 (2.5%)		5 (3.8%)
**Education level**			
High school diploma/GED	2 (0.8%)		0
Trade school/Vocational school	1 (0.4%)		0
(Some) College	18 (7.5%)		6 (4.5%)
College graduate	74 (30.8%)		43 (32.3%)
Graduate school/professional school	143 (59.6%)		83 (62.4%)
Declined to respond	2 (0.8%)		1 (0.8%)
**Current Working Position**^a^			
Faculty physician	38 (15.8%)		16 (12.0%)
Resident physician	49 (20.4%)		44 (33.1%)
Physician assistant	12 (5.0%)		6 (4.5%)
Nurse practitioner	7 (2.9%)		2 (1.5%)
Licensed registered nurse	79 (32.9%)		48 (36.1%)
Social worker	1 (0.4%)		0
Other	54 (22.5%)		17 (12.8%)
**Years in current position**^a^	6.0 (6.6)^h^		4.4 (5.2)^i^
**Assessments**	278		168
**MENTAL HEALTH STATUS**			
**Resilience**^**b**^	15.0 (2.3)		15.3 (2.2)
**Burnout**^**c**^			
Personal Accomplishment	13.4 (3.2)		13.4 (3.2)
Emotional Exhaustion	9.7 (4.6)		9.9 (4.6)
Depersonalization	5.9 (4.5)		6.4 (4.5)
*Probable burnout (if EE* ≥ *11 and DEP* ≥ *7)*	89 (32.0%)		57 (33.9%)
**Depressive symptoms**^**d**^	5.1 (4.4)		5.6 (4.6)
*Probable depression (cut-off* ≥ *20)*	2 (0.7%)		1 (0.6%)
**Anxiety symptoms**^**e**^	5.1 (4.6)		5.4 (4.7)
*Probable anxiety (cut-off* ≥ *15)*	13 (4.7%)		10 (6.0%)
**PTSD symptoms**^**f**^			
Total score	15.5 (13.1)		15.9 (13.0)
Cluster B Intrusion	3.8 (3.8)		3.8 (3.6)
Cluster C Avoidance	1.8 (2.0)		1.9 (2.0)
Cluster D Negative Cognitions and Mood	5.4 (5.3)		5.6 (5.4)
Cluster E Arousal and Reactivity Alterations	4.4 (4.1)		4.6 (4.2)
*Probable PTSD (cut-off* ≥ *31)*	43 (15.5%)		28 (16.7%)

Scores are presented for the participant sample used for the LPA analysis and the sub selection of participants used for the prediction analysis (aka those whose video data were available to extract temporal features as digital biomarkers). Sex at birth was based on self-reports. Scores displayed as mean (SD) for continuous variables or n(%) for categorical variables.

^a^Measured during baseline assessment.

^b^Measured with total scores on the Brief Resilience Coping Scale (BRCS, range 4–20).

^c^Measured with domain scores on the Maslach Burnout Inventory (MBI-9, range 0–18).

^d^Measured with total scores on the Patient Health Questionnaire (PHQ-8, range 0–24).

^e^Measured with total scores on the General Anxiety Disorder (GAD-7, range 0–21).

^f^Measured with total and subdomain scores on the PTSD Checklist for DSM-5 (PCL-5, range 0–80).

^g^Information for n = 2 participants missing.

^h^Information for n = 6 participants missing.

^i^Information for n = 1 participant missing. Sensitivity analysis evaluating potential sources of sampling and demographic bias across sample pools are presented in Supplementary Material S7.

##### Timeseries construction and temporal feature extraction

To capture dynamic changes and temporal variation in facial expressivity throughout the full video-recorded semi-structured interview, we extracted a set of temporal facial features based on previous research that identified these as relevant markers sensitive to changes in emotional responsivity and internal affective state dynamics [50]. We constructed frame-level time series that capture multiple facial signal types using Python (v3.9). First, timeseries of emotion classification probabilities (0–1) were estimated for seven emotions per frame, including anger, disgust, fear, sadness, happiness, surprise, and neutral, using Py-Feat (v1.0.1). Probabilities were set to zero in case of low-confidence predictions (< 0.60). In addition, emotion polarity was calculated to assess the relative probability of expressing positive over negative emotions (−1 to +1), by subtracting the probabilities of negative emotions (sum of the probabilities for anger, sadness, fear, and disgust) from positive (happiness) per frame. After calculating emotion polarity scores, we applied a threshold of −0.3–+0.3 to bin them into three categories: positive (emotion polarity > +0.3), negative (emotion polarity < −0.3) and neutral (emotion polarity between −0.3 and +0.3). Timeseries of activated facial action units (AUs) were also estimated (0 or 1) per frame using Py-Feat, which is defined as a facial movement when emotions are expressed (e.g., raising eyebrows, smiling). Due to the computational load of Py-Feat, we subsampled the video segments and processed every third frame to construct the timeseries. In addition, time series of the intensity of the activated facial AU (0–1) were constructed per frame using OpenFace (v2.0.0). To reduce motion artifacts arising from speaking, only upper-face AUs were included. Down-sampling effects were mitigated by applying Gaussian noise filtering. Valence and arousal levels (−1 to 1) were estimated per frame using FaceTorch (v0.5.1). Importantly, frames were processed only if they contained a visible face, which was decided based on a predefined face detection threshold (Face Detection Confidence Scores for Py-Feat ≥ 97; OpenFace>0.88). To ensure comparability across participants, the number of frames within each timeseries was set to be the minimum number of valid frames across our dataset.

Lastly, we constructed a set of summary statistics representative of these dynamic timeseries (i.e., emotion duration and transition counts) and timeseries characteristics (i.e., changepoint detection (Ruptures package, v1.1.9) and peak detection). See Supplementary Material S4 for an overview of all facial features.

#### Machine learning model

To classify ED HCWs into the latent profiles based on their facial features, we implemented a robust machine learning (ML) pipeline in Python (v3.9, scikit-learn v1.5.2). We applied supervised ML models prioritizing interpretability and clinical relevance while enabling non-linear associations between the features. The classification approach was structured around a nested cross-validation framework to ensure unbiased estimation of model performance, validate model robustness, and prevent information leakage across training and test folds. Each iteration consisted of an outer 5-fold cross-validation loop for model evaluation and an inner 5-fold cross-validation loop for hyperparameter optimization and feature selection. Within the inner loop, a grid search was applied to maximize the F1-score. All features were scaled individually using Min-Max Normalization, normalizing feature values to a range of 0–1. Scaling parameters were fit exclusively on the training data within each fold and applied to the corresponding test data. For dimensionality reduction, we applied Recursive Feature Elimination with Cross-Validation (RFECV) using a linear Support Vector Classifier (SVC) estimator within the inner loop, enforcing a minimum of 10 informative features. We used both class weighting and Synthetic Minority Oversampling Technique (SMOTE) due to the class imbalance between the two latent profiles. All preprocessing steps, including feature scaling, oversampling, and feature selection, were performed strictly within the training data of each cross-validation fold to prevent data leakage. Oversampling was applied only within the training folds and preserved participant-level independence. The full pipeline, including model training and prediction, was executed independently within each fold.

Four classifiers were compared: Logistic Regression with L1 regularization, Logistic Regression with Elastic-net regularization, Support Vector Classification (SVC) with multiple kernels (linear, poly, rbf, sigmoid), and eXtreme Gradient Boosting (XGBoost). These classifiers were selected for balancing model complexity with result interpretability. We additionally incorporated a soft-voting ensemble (VotingClassifier) that aggregated probabilistic predictions from all base classifiers by averaging their predicted class probabilities and selecting the class with the highest mean probability. Combining probabilistic outputs leverages complementary decision boundaries and stabilizes predictions, improving robustness by reducing model-specific biases and variance. For each classifier, performance was assessed using Precision, Recall, F1-score, and Area Under the Receiver Operating Characteristics Curve (AUROC), aggregating these metrics across outer folds to report the mean and standard deviation. Final performance metrics were calculated only on the held-out test sets from the outer cross-validation loop.

To interpret model decisions, we employed SHapley Additive exPlanations (SHAP [51]). SHAP values were computed to quantify the contribution of each facial feature to the predicted class probability.

## Results

### Clinical phenotypes

**Fig. 1:**
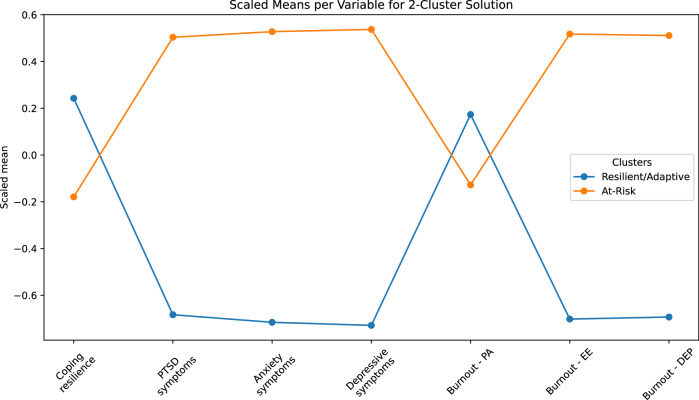
Latent profiles of the At-risk and Resilient/Adaptive Phenotypes. A visualization of the latent profiles for the Resilient/Adaptive Phenotype (42.4%, 118 assessments in n = 105 participant) and At-Risk Phenotype (57.6%, 160 assessment in n = 140 participants). Profiles are based on total and subdomain scores for resilient coping (BRCS), burnout (MBI-9), depression (PHQ-8), anxiety (GAD-7) and PTSD (PCL-5). Scores are scaled mean scores. PA: personal accomplishment, EE: emotional exhaustion, DEP: Depersonalization.

LPA was performed for 278 assessments from N = 240 participants (Table 1). The best-fitting LPA model consisted of two profiles according to best balance between model fit, classification quality, and interpretability (Fig. 1, Table 2, Supplementary Material S5). Based on means of total and subdomain scores, we labeled the descriptive profiles “At-risk Phenotype” and “Resilient/Adaptive Phenotype”. The “At-risk Phenotype” (57.6%, 160 assessments, n = 140 participants) was characterized by significantly higher symptom levels for burnout-related emotional exhaustion and depersonalization, depression, anxiety, and PTSD, and lower resilient coping and burnout-related personal accomplishment levels. The “Resilient/Adaptive Phenotype” (42.4%, 118 assessments from n = 105 participants) was characterized by significantly lower symptom levels for burnout-related emotional exhaustion and depersonalization, depression, anxiety, and PTSD, and higher resilient coping and burnout-related personal accomplishment levels.

**Table 2:** Estimated mean total- and subdomain scores of the At-risk and Resilient/Adaptive Phenotypes of the best-fitting model with regards to mental health for the latent profile analysis sample.

	Phenotypes		
	At-Risk Phenotype	Resilient/ Adaptive Phenotype	Statistics (two-tailed)
Participants	140	105	
**Assessments**	160 (57.6%)	118 (42.4%)	
**MENTAL HEALTH STATUS**			
**Resilience**^**a**^	14.6 (2.1)	15.5 (2.6)	F(1 271.15) = 8.73, *p* = 0.003*
**Burnout**^**b**^			
Personal Accomplishment	13.0 (3.2)	13.9 (3.0)	F(1 272.47) = 5.77, *p* = 0.017*
Emotional Exhaustion	12.1 (3.5)	6.5 (3.9)	F(1 274.95) = 149.66, *p* < 0.001*
Depersonalization	8.2 (4.2)	2.8 (2.6)	F(1 266.64) = 128.36, *p* < 0.001*
*Probable burnout (if EE* ≥ *11 and DEP* ≥ *7)*	84 (52.5%)	5 (4.2%)	
**Depressive symptoms**^**c**^	7.5 (4.3)	1.8 (1.7)	F(1 269.97) = 156.19, *p* < 0.001*
*Probable depression (cut-off* ≥ *20)*	2 (1.3%)	0	
**Anxiety symptoms**^**d**^	7.5 (4.5)	1.8 (1.8)	F(1 313.98) = 150.26, *p* < 0.001*
*Probable anxiety (cut-off* ≥ *15)*	13 (8.1%)	0	
**PTSD symptoms**^**e**^			
Total score	22.1 (13.3)	6.5 (5.0)	F(1 277.33) = 126.71, *p* < 0.001*
Cluster B Intrusion	5.3 (4.2)	1.8 (1.8)	
Cluster C Avoidance	2.5 (2.2)	0.8 (1.1)	
Cluster D Negative Cognitions and Mood	7.9 (5.5)	2.0 (2.3)	
Cluster E Arousal and Reactivity Alterations	6.3 (4.4)	1.9 (1.8)	
*Probable PTSD (cut-off* ≥ *31)*	43 (26.9%)	0	

^a^Measured with total scores on the BRCS: Brief Resilience Coping Scale, range 4–20.

^b^Measured with domain scores on the MBI-9: Maslach Burnout Inventory, range 0–18.

^c^Measured with total scores on the PHQ-8: Patient Health Questionnaire, total range 0–24.

^d^Measured with total scores on the GAD-7: General Anxiety Disorder-7, total range 0–21.

^e^Measured with total and subdomain scores on the PCL-5: PTSD Checklist for DSM-5, total range 0–80. For post hoc test results, effect sizes and confidence intervals, see Supplementary Material S5.

### Model performance and explainability

**Fig. 2:**
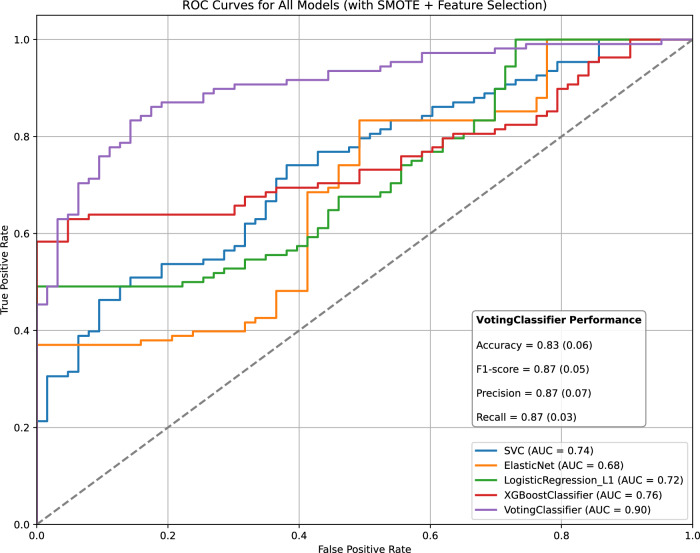
AUROC curves for all classification ML models. AUROC curves in the test set for all classification ML models. The Area Under the Receiver Operating Characteristics (AUROC) for all classifier ML models (logistic regression with L1 regularization model, logistic regression with elastic net regularization model, SVC model, XGBoost model, and VotingClassifier) in the test set. For each model, the AUROC was created by taking all the prediction probabilities on the test set across the folds.

In the subset of 168 video recordings of completed interviews from N = 133 participants, both the logistic regression with elastic net regularization and SVM consistently achieved the highest performance on both train and test folds, indicating satisfactory generalization. The VotingClassifier, combining probabilistic predictions from all base classifiers, achieved the highest AUROC score (0.90), suggesting that all classifiers combined can robustly distinguish between the phenotypes using the temporal dynamic facial features (Fig. 2, Supplementary Material S6).

**Fig. 3:**
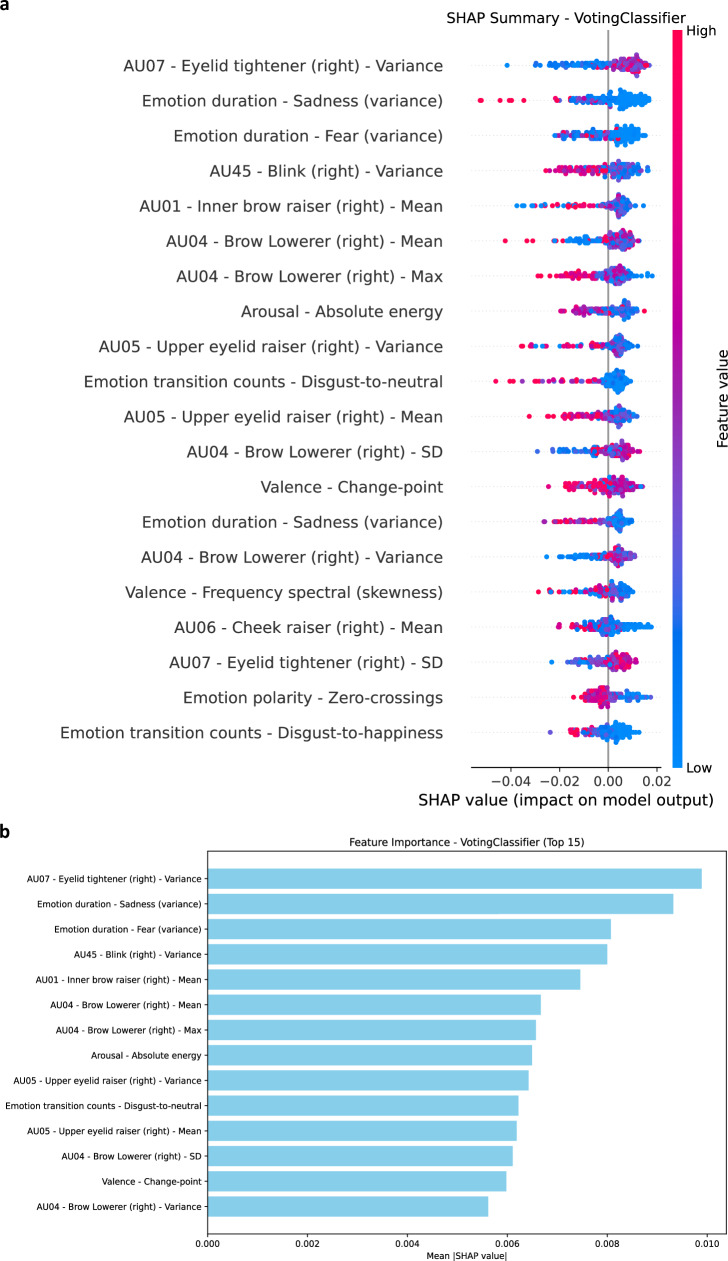
SHAP summary plots. **a** SHAP summary plot in the VotingClassifier for predicting the At-risk and Resilient/Adaptive Phenotypes. **b** Feature importance summary plot in the VotingClassifier for predicting the At-risk and Resilient/Adaptive Phenotypes. Greater mean SHAP values indicate higher feature importance to the model. Each plot illustrates how individual features impact the model’s prediction, with each point representing a single participant. The horizontal position of a point reflects its influence on the prediction: values on the left of the axis (below 0) correspond to the Resilient/Adaptive phenotype, while those on the right (above 0) correspond to the At-risk phenotype. Points further to the left or right signify a stronger contribution to the phenotypes. The color gradient represents the original value of the feature, with higher values shown in pink and lower values in blue.

The SHAP results present a clear pattern of features that contribute to predictions of the latent phenotypes (Fig. 3). The variance in intensity of the right AU7 (Lid Tightener) and variance in duration of expressing sadness and fear are identified as the most significant features for classifying individuals into the at-risk phenotype.

Sensitivity analyses evaluating potential sources of sampling and demographic bias are presented in Supplementary Material S7 and provided no evidence that sample selection due to video availability or demographic characteristics, including age and race, influenced the ML results.

## Discussion

In this study, using passively observed video data from stress-inducing interviews in ED HCWs, we captured objective temporal patterns of facial expressivity as behavioral indicators of expressive flexibility, which has been linked to resilience and identified as a protective factor against mental health risk. Using an ensemble VotingClassifier model, we found that individuals showing greater expressive flexibility, reflected by more dynamic and variable facial expressivity over time throughout predominantly affective states, were more likely to be classified into the resilient/adaptive phenotype. By capturing temporal fluctuations in facial expressions, rather than solely relying on static measures per singular timepoint, our approach provides insights into how individuals respond to stressors in real time. Our findings highlight the feasibility and clinical potential of digital biomarkers from facial dynamics derived from passively observed video data as unobtrusive and objective proxies of subtle adaptive emotional responding in high-stress contexts. Such approach could complement and improve early detection and identification of mental health risk among ED HCWs, particularly in stigma-sensitive settings. While further validation is needed to establish clinical utility, this work highlights the potential of digital phenotyping to inform resilience-focused approaches and how expressive flexibility related to HCW’s capacity to manage emotional demands to enhance resilience, mental health monitoring, and prevention.

Our LPA identified two clinically relevant latent phenotypes characterized by differential symptomatology: an at-risk phenotype, characterized by high psychological symptoms and relatively lower resilient coping; and a resilient/adaptive phenotype, showing the opposite pattern. Our sample included a large proportion of HCWs reporting elevated psychological symptoms (58%) as well as high prevalence of probable burnout (32.0 and 33.9% in the respective sample pools) and PTSD (15.5 and 16.7%), which is comparable with previous studies that found a high burden in healthcare professionals [52, 53]. In addition, our assessments were conducted during and after the COVID-19 pandemic, which may have contributed to high symptom levels. Increased awareness of mental health issues and reduced stigma in the post-pandemic period could also have encouraged more open self-reporting among HCWs. Furthermore, our person-centered LPA approach, which integrates a comprehensive range of mental health outcomes, likely provides a more nuanced and less biased objective representation of psychological well-being compared to single-outcome or resilient-only assessments. This transdiagnostic approach seems particularly relevant among HCWs, where symptoms of PTSD, depression, anxiety, and burnout frequently co-occur and may reflect shared responses to chronic occupational stress [54–57]. Rather than identifying diagnosis-specific markers, our aim was to detect individuals exhibiting a broader pattern of elevated psychological risk who may benefit from further assessment or intervention. This approach also aligns with growing interest in transdiagnostic models of psychopathology, which emphasize broader dimensions of psychological vulnerability that cut across traditional diagnostic boundaries [58, 59].

Our ML results seem to support prior evidence linking the ability to flexibly adjust and adaptively upregulate and downregulate emotional responses to contextual demands to lower distress and improved adjustment under chronic stress [33, 60, 61]. Findings additionally align with Bonanno’s theory of resilience, which conceptualizes adaptation as a dynamic process of flexible self-regulation [14, 62], in which expressive flexibility is a central mechanism that enables individuals to ‘bounce back’ and adaptively recover after stress exposure [12, 13, 25]. Individuals showing more nuanced and adaptable expressive patterns were less likely to fall into at-risk trajectories for poor mental health, which may be indicative of a broader repertoire of expressive responses and the capacity to adjust their behavior over time based on situational feedback. This supports expressive flexibility as a behavioral indicator of protective processes. Although other digital biomarkers such as specific facial action units also contributed to the prediction of the resilient/adaptive and at-risk phenotypes, their patterns were less clearly defined than those observed for affective states and warrant further investigation. Our results suggest that dynamic facial expressivity may serve as a clinically meaningful proxy of resilience-related processes, reflecting the potential capacity to recover or ‘bounce back’ from stress, providing empirical support for connecting moment-to-moment dynamic expressivity to broader constructs of adaptive emotion regulation and resilience.

Our study contributes to the growing field of digital phenotyping [43, 63] by demonstrating that digital biomarkers of dynamic facial expressivity can serve as an objective, scalable proxy of behavioral patterns associated with resilience-related phenotypes and mental health risk in high-stress clinical environments. Using advanced computer vision, we offered an automated, unobtrusive approach to quantifying expressive behavior in naturalistic contexts using passive video data. Such technology-enabled assessments may hold particular promise in stigma-rich settings, where traditional self-reports and clinically administered tools may be underutilized or biased by underreporting and stigma [8–10, 37], and can be complemented by objective behavioral information. Their application in workplace settings also raises important considerations regarding privacy, autonomy, and potential misuse, underscoring the need for careful and ethical implementation. Importantly, expressive flexibility represents only one facet of resilience and integrating multimodal indicators across multiple domains, such as behavioral, biological, and self-reported components, could yield a more comprehensive understanding of adaptive capacity and improve prediction of risk and resilience trajectories [64].

As objective features of expressive behavior were associated with the resilient/adaptive phenotype in our study, our approach may offer a scalable, low-burden method for early risk detection [65–67], especially since facial dynamics can be measured passively through widely accessible digital platforms. Notably, the present findings were obtained in a racially diverse HCWs sample, and sensitivity analyses did not identify significant differences in model prediction probabilities across racial groups (Supplementary Material S7). These findings suggest that model outputs were broadly comparable across the racial groups represented in the study and provide preliminary support for the applicability of this approach across diverse healthcare worker populations. Integrating digital biomarkers of expressive flexibility could guide future studies examining how HCWs manage emotional demands. This could ultimately inform and potentially complement existing resilience-building programs, such as those targeting emotion regulation training, cognitive reappraisal, mindfulness-based approaches, and flexible coping skills [68, 69]. Our findings highlight a promising first step towards multimodal, precision-based mental health and resilience identification in high-stress professions and support the future potential for digital phenotyping to support early identification and prevention efforts.

Several limitations should be considered when interpreting findings. First, clinical phenotypes were derived from self-reported measures and models predicted these symptom-based phenotypes, which may limit the accuracy of our phenotypes and classification due to potential underreporting and subjective bias [8, 9]. We partially mitigated these biases by integrating several validated mental health measures and using LPA to derive more stable clinical phenotypes. From a methodological perspective, facial expressivity was used as a behavioral proxy of adaptive processes and does not fully capture the complexity of internal emotion regulation processes. Yet, it offers valuable insights into dynamic patterns of externally observable responses to stressors in real-world contexts. Although more fine‑grained sequence‑based models could capture higher‑order temporal dependencies, we intentionally chose this approach to provide theoretically grounded and interpretable proxies of expressive flexibility. Building on this, integrating multimodal digital biomarkers, including biological indices of chronic stress, auditory and speech-based features, and subjective measures of emotion regulation, may enable more comprehensive models of adaptive functioning.

Temporal dynamics in facial expressivity may offer a valuable perspective into how individuals respond related to emotion regulation processes, mental health risk, and resilience. By linking expressive behavior to resilience-related phenotypes, our findings support the potential of computational approaches within the emerging field of digital phenotyping to provide markers that are accurate, scalable, personalized, and grounded in ecological-valid behavioral for mental health risk. Ultimately, our study may guide future studies examining expressive flexibility and could potentially inform the next generation of resilience and wellness programs, complementing existing emotion regulation-focused interventions to enhance psychological well-being and resilience in high-stress professional settings.

### Citation diversity statement

The authors have attested that they made efforts to be mindful of diversity in selecting the citations used in this article.

## Supplementary information

Supplementary Material - Bounce Back

## Data Availability

The dataset generated and analyzed during as well as the programming code used for the current study are not publicly available but are available from the corresponding author on reasonable request.
