# Supplementary material for: Bouncing back from stress: objective markers of expressive flexibility and resilience in emergency healthcare workers using computer vision
Source: NPP Digit Psychiatry Neurosci. 2026 Jul 15;4:16. doi: 10.1038/s44277-026-00067-y (PMC13369946; doi:10.1038/s44277-026-00067-y)
Supplement: Supplementary file 1 — Supplementary Material - Bounce Back [file 44277_2026_67_MOESM1_ESM.pdf]

S1. Data availability flowchart. Each assessment is treated as a separate observation for data analysis.

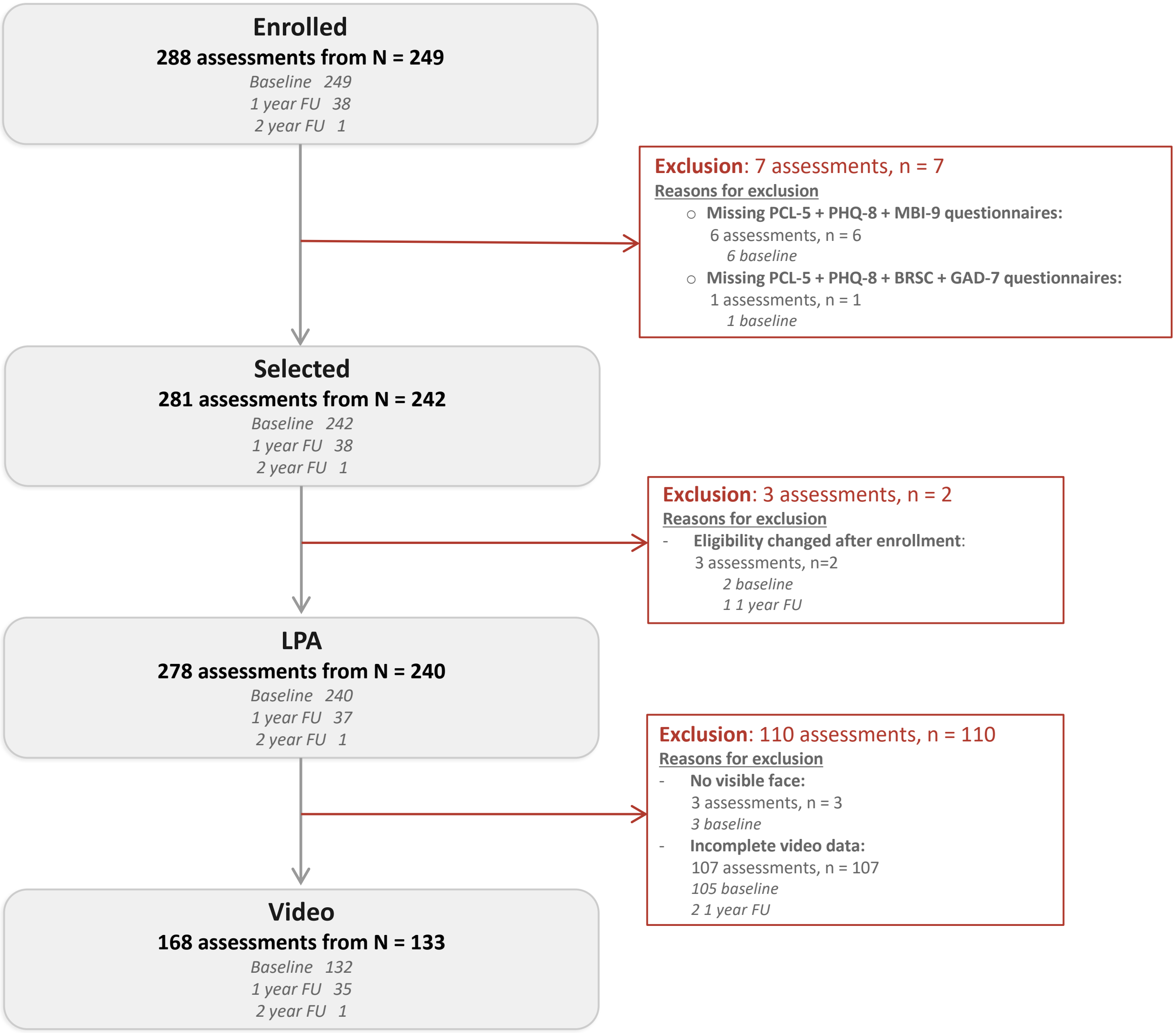

FU: Follow-up; PCL-5; PTSD Checklist for DSM5; PHQ-8: Patient Health Questionnaire; MBI-9: Maslach Burnout Inventory; BRCS: Brief Resilience Coping Scale; GAD-7: General Anxiety Disorder-7.

S2.1 Latent Profile Analyses (LPA) preprocessing and best-fitting model selection.

Feature values included in the latent profile analysis (LPA) were BRCS, PHQ-8, GAD-7, and PCL-5 sum scores and MBI-9 PA, EE, and DEP subdomain scores, which were first standardized by centering them around the mean and scaling to unit variance before performing LPA. Latent profile model evaluation was based on pre-identified indicators of the best-fitting model by comparing the fit of a k-profile model to a subsequently k+1-profile model until the most optimal model was found [1]. These indicators included the Bayesian Information Criterion (BIC), Akaike

Information Criterion (AIC), adjusted-AIC (lower values defining better model fit), and entropy (>.80 defining adequate profile division [2]. To avoid the bias of reaching local maxima, models were estimated with multiple initial random starts and final stage optimizations [3].

S2.2 Results of Latent Profile Analyses (LPA) on best-fitted classification model using pre-identified criteria and selection indicators.

	1-profile model	2-profile model	3-profile model
**N=278**			
**LL**	-2 432.308	-2 289.803	-2 229.015
**AIC**	4 934.616	4 721.605	**4 672.055**
**BIC**	5 061.583	**4 979.166**	5 060.211
**SS Adjusted-BIC**	4 950.602	4 754.033	**4 720.926**
**Entropy**	-	**0.971**	0.964

AIC: Akaike Information Criterion; BIC: Bayesian Information Criterion; SS Adjusted-BIC: Sample Size Adjusted BIC; -LL: Log-likelihood.

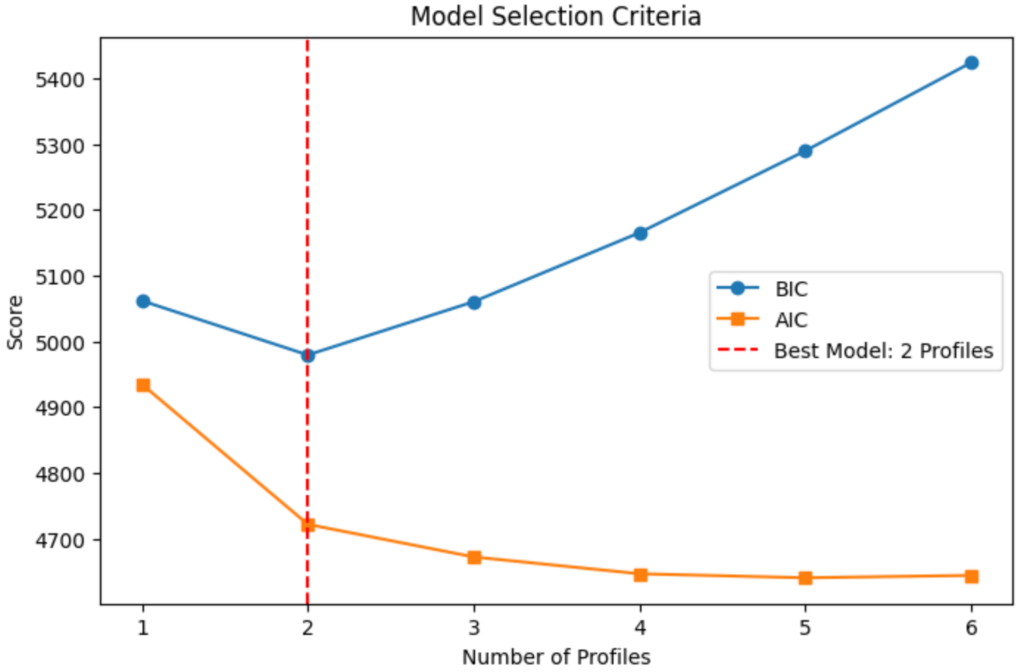

S2.3 Final model selection and latent symptom profiles.

Model indicators were compared across 1-, 2-, 3-, 4-, 5-, and 6-profile models. However, increasing the number of profiles to a 3-profile model and higher resulted in reduced model reliability based on the criteria values, with less reliable classification quality and diminished gains based on reduced balance between fit and parsimony. In comparison with a 1-profile model, inclusion of 2 profiles improved the model, accompanied by the lowest BIC and highest entropy values in the 2-profile model across all models[1, 4], indicating clear division and classification

quality between 2 latent profiles. In addition, the 2-profile model resulted in the largest decrease in AIC values (-4.3%) and SS Adjusted-BIC (-4.0%) compared to the other *k*+1 models. With inclusion of a 3- over a 2-profile model, BIC increased by 1.6%, entropy decreased by 0.01%, and there was only a slight decrease in AIC (-1.0%) and SS Adjusted-BIC (-0.01%) values. For all k-profile models, classification quality entropy statistics were considered adequate (p>0.964). In consideration of all indicators, the 2-profile model was likely the most meaningful fitted model with acceptable interpretability and high classification precision.

S3. Sensitivity analysis multivariate outliers

We performed a sensitivity analysis in which we excluded N=10 participants with 11 assessments due to being multivariate outliers on mental health symptom scores (Mahalanobis Distances), resulting in a total of N = 233 participants with 266 assessments. Differences on continuous data for mental health status between final profiles were assessed using Linear Mixed Models (LMM) with Restricted Maximum Likelihood (REML) in SPSS 28.0.1.1 (IBM SPSS Statistics Software). Best-fitted model with First-Order Autoregressive covariance structure had lowest values for -2 Log Likelihood, AIC and BIC criteria compared to models with Unstructured or first-order autoregressive covariance structure.

S3.1 Sensitivity analysis: Results of Latent Profile Analyses (LPA) on best-fitted classification model using pre-identified criteria and selection indicators.

	**1-profile model**	**2-profile model**	**3-profile model**	**4-profile model**	**5-profile model**
**N=233**					
**LL**	-2 333.750	-2 213.161	-2 150.883	-2 109.766	-2 067.623
**AIC**	4 737.500	4 568.322	**4 515.765**	4 505.532	4 493.259
**BIC**	4 863.185	**4 823.282**	4 900.001	5 019.043	5 136.046
**SS Adjusted-BIC**	4 752.213	4 598.168	**4 560.745**	4 565.645	4 568.505
**Entropy**	-	**0.969**	0.964	0.959	0.958

AIC: Akaike Information Criterion; BIC: Bayesian Information Criterion; -2LL: -2 times log-likelihood difference between a *k*-class solution and *k*+1-class solution.

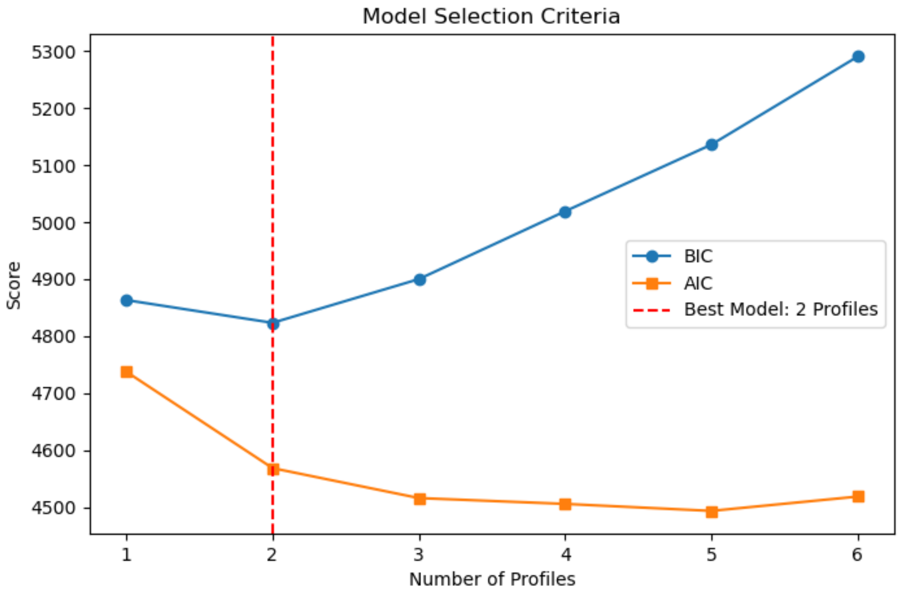

S3.2 Sensitivity analysis: Final model selection and latent symptom profiles.

Results on model indicators and evaluation were similar to the original LPA, with increasing the number of profiles to a 3-profile model and higher resulting in reduced model reliability based on the criteria values, with less reliable classification quality and diminished gains based on reduced balance between fit and parsimony. In comparison with a 1-profile model, inclusion of 2 profiles improved the model, accompanied by the lowest BIC and highest entropy values in the 2-profile model across all models, indicating clear division and classification quality between 2 latent profiles. In consideration of all indicators, the 2-profile model was likely the most meaningful fitted model with acceptable interpretability and high classification precision.

S3.3 Sensitivity analysis: Estimated mean total- and domain scores of the mental health profiles of the best-fitting model with regards to mental health for the clustering sample without outliers.

**N=233**	**Phenotypes**		
	**At-Risk Phenotype**	**Resilient/ Adaptive Phenotype**	**Statistics** (two-tailed)
**Assessments**	147 (54.9%)	121 (45.1%)	
**MENTAL HEALTH STATUS**			
**Resilience**[1]	14.8 (2.1)	15.5 (2.2)	F(1,265.91)=6.15, *p*=.014*
**Burnout**[2]			
Personal Accomplishment	13.2 (2.9)	14.1 (2.9)	F(1,262.99)=4.45, *p*=.036*
Emotional Exhaustion	12.0 (3.5)	6.9 (4.0)	F(1,264.23)=126.35, *p*<.001*
Depersonalization	7.4 (4.1)	4.2 (4.1)	F(1,254.04)=35.79, *p*<.001*
Probable burnout (if EE ≥ 11 and DEP ≥ 7)	69 (46.9%)	18 (14.9%)	
**Depressive symptoms**[3]	7.5 (3.9)	1.8 (1.7)	F(1,264.86)=211.29, *p*<.001*
Probable depression (cut-off ≥ 20)	0	0	
**Anxiety symptoms**[4]	7.6 (4.0)	1.8 (1.8)	F(1,50.99)=279.58, *p*<.001*
Probable anxiety (cut-off ≥ 15)	10 (6.8%)	0	
**PTSD symptoms**[5]			
Total score	23.3 (11.4)	5.6 (4.0)	F(1,261.33)=236.97, *p*<.001*
Cluster B Intrusion	5.7 (3.9)	1.4 (1.5)	
Cluster C Avoidance	2.7 (2.1)	0.7 (1.0)	
Cluster D Negative Cognitions and Mood	8.3 (4.9)	1.8 (1.9)	
Cluster E Arousal and Reactivity Alterations	6.6 (3.9)	1.7 (1.7)	
Probable PTSD (cut-off ≥ 31)	40 (27.2%)	0	

[1]BRCS total scores: Brief Resilience Coping Scale, range 4-20; [2]MBI-9 domain scores: Maslach Burnout Inventory, range 0-18; [3]PHQ-8 total scores: Patient Health Questionnaire, total range 0-24; [4]GAD-7 total scores: General Anxiety Disorder-7, total range 0-21; [5]PCL5 total and subcluster scores: PTSD Checklist for DSM5, total range 0-80.

S3.4 Post hoc test results of Linear Mixed Models (LMM) with First-Order Autoregressive covariance structure for mental health measures that differed significantly between the At-Risk and Adaptive/Resilient Phenotypes, excluding outliers of N=10 participants with 11 assessments.

	**Fixed Effects of Profiles** (two-tailed)				**Post hoc test statistics and interpretation**
	**Numerator df**	**Denominator df**	**F**	***p***	High-Symptom vs. Low-Symptom Phenotypes
**Resilience[1]**	1	265.91	6.15	*.014*	Resilience scores were lower in the At-Risk (14.75 (0.18)) than Adaptive/Resilient Phenotype (15.39 (0.20)) *B* = -0.64, 95% CI -1.15 to -0.13, SE = 0.26, *t* = -2.48, *p*=.014, Cohen's f^2 = 0.03
**Burnout[2]** Personal Accomplishment (PA)	1	262.99	4.45	*.036*	Personal accomplishment scores were lower in the At-Risk (13.20 (0.24)) than Adaptive/Resilient Phenotype (13.93 (0.27)) *B* = -0.74, 95% CI -1.64 to -1.42, SE = 0.05, *t* = -2.11, *p*=.036, Cohen's f^2 = 0.03
Emotional Exhaustion (EE)	1	264.23	126.35	*<.001*	Emotional exhaustion scores were higher in the At-Risk (11.96 (0.32)) than Adaptive/Resilient Phenotype (6.84 (0.35)) *B* = 5.12, 95% CI 4.22 to 6.01, SE = 0.46, *t* = 11.24, *p*<.001, Cohen's f^2 = 0.53
Depersonalization (DEP)	1	254.04	35.79	*<.001*	Depersonalization scores were higher in the At-Risk (7.20 (0.34)) than Adaptive/Resilient Phenotype (4.33 (0.37)) *B* = 2.87, 95% CI 1.92 to 3.81, SE = 0.48, *t* = 5.98, *p*<.001, Cohen's f^2 = 0.15
**Depressive symptoms[3]**	1	264.86	211.29	*<.001*	Depressive symptom scores were higher in the At-Risk (7.33 (0.26)) than Adaptive/Resilient Phenotype (1.86 (0.29)) *B* = 5.47, 95% CI 4.73 to 6.21, SE = 0.38, *t* = 14.54, *p*<.001, Cohen's f^2 = 0.89
**Anxiety symptoms[4]**	1	50.99	279.58	*<.001*	Anxious symptom scores were higher in the At-Risk (7.44 (0.24)) than Adaptive/Resilient Phenotype (1.74 (0.26)) *B* = 5.69, 95% CI 5.01 to 6.38, SE = 0.34, *t* = 16.72, *p*<.001, Cohen's f^2 = 1.21
**PTSD symptoms[5]**	1	261.33	236.97	*<.001*	PTSD symptom scores were higher in the At-Risk (22.68 (0.75)) than Adaptive/Resilient Phenotype (6.19 (0.79)) *B* = 16.48, 95% CI 14.37 to 18.59, SE = 1.07, *t* = 15.39, *p*<.001, Cohen's f^2 = 1.02

Test statistics are presented as mean (SD). Q[1]BRCS total scores: Brief Resilience Coping Scale, range 4-20; [2]MBI-9 domain scores: Maslach Burnout Inventory, range 0-18; [3]PHQ-8 total scores: Patient Health Questionnaire, total range 0-24; [4]GAD-7 total scores: General Anxiety Disorder-7, total range 0-21; [5]PCL5 total and subcluster scores: PTSD Checklist for DSM5, total range 0-80. Cohen's f^2 effect sizes were calculated following guidelines of GroB & Möller[5].

S4. Overview and description of timeseries and temporal dynamic facial features.

Timeseries	Feature Description
**Emotion classification probability** (0-1)<br>1. Anger<br>2. Disgust<br>3. Fear<br>4. Sadness<br>5. Happiness<br>6. Surprise<br>7. Neutral	Probability of expressing a specific emotion from the full face within seven different emotion classifications.<br>Package used: Py-Feat (v1.0.1 [6, 7]).
**Emotion polarity** (-1 to +1)	Relative probability of expressing positive over negative emotions.<br>= (Emotion classification probability Happiness – (Emotion classification probabilities Anger + Sadness + Fear + Disgust))<br><br>- Positive emotion polarity: >+0.3<br>- Negative emotion polarity: <-0.3<br>- Neutral emotion polarity: between -0.3 and +0.3
**Activated facial action units** (0 or 1)	Activation of 7 facial action units across the upper-face, which indicates facial movement when emotions are expressed, e.g. raising eyebrows, etc.<br>Package used: Py-Feat (v1.0.1 [6, 7]).
**Facial action unit intensities** (0-1)	The intensity of each activated facial action across the upper-face.<br>Package used: OpenFace (v2.0.0 [8]).
**Valence and Arousal signal levels** (-1 to 1)	The level of valence and arousal from the full face.<br>Package used: FaceTorch (v0.5.1 [9]).

Temporal Dynamic Facial Features	Timeseries used	Feature Description
**Changepoint detection**	Valence signal levels,<br>Arousal signal levels,<br>Emotion polarity	The number of moments when an emotional classification shifts from one to the other, which indicates consistency of emotion expressiveness as well as the frequency of transitioning from one emotional state to another.<br><br>A higher number of changepoints may reflect more shifts in expressed emotions. A lower number of changepoints may reflect dampening or less shifts in expressed emotions.

**Emotion duration**	7 Emotion classifications	The duration of expressing one single emotion before transitioning to another emotion.<br><br>A longer duration may reflect stability in the expression of an emotion. A shorter duration may indicate quick flexible expressions of emotions.
**Emotion transition counts**	7 emotion classifications, Emotion polarity	1. The number of transitions between each emotion classification.<br>2. The number of transitions between each emotion polarity status (positive, negative, neutral).<br><br>This may provide a proxy for *Emotional Agility*—the capacity to navigate between different emotions or emotional states. More variability and balanced transitions between positive and negative states may reflect adaptive and flexible transitions in emotion expressivity, whereas unidirectional or stagnant patterns might suggest less flexible transitions in emotion expressivity.
**Peak detection**	Valence signal levels, Arousal signal levels, Emotion polarity	Number of positive and negative peaks for each timeseries.<br><br>A higher number of peaks indicates a higher frequency of expressing a specific emotion or emotional state.
**Summary statistics of timeseries dynamics**	Valence signal levels, Arousal signal levels, Emotion polarity	Statistics of timeseries dynamics that can characterize the rhythm and intensity of emotion expressivity. For example, FFT feature, zero-crossing, and absolute energy. This offers insights into the overall dynamic and stability of emotion expressivity.

S5. Post hoc test results of Linear Mixed Models (LMM) with First-Order Autoregressive covariance structure for mental health measures that differed significantly between the At-Risk and Adaptive/Resilient Phenotypes.

	**Fixed Effects** (two-tailed)				**Post hoc test statistics and interpretation**
	**Numerator df**	**Denominator df**	**F**	***p***	At-Risk vs. Adaptive/Resilient Phenotype
**Resilience**[1]	1	271.15	8.73	*.003*	Resilience scores were lower in the At-Risk (14.53 (0.19)) than Adaptive/Resilient Phenotype (15.37 (0.22)) *B* = -0.83, 95% CI -1.39 to -0.28, SE = 0.28, *t* = -3.00, *p*=.003, Cohen's f^2 = 0.03
**Burnout**[2] Personal Accomplishment (PA)	1	272.47	5.77	*.017*	Personal accomplishment scores were lower in the At-Risk (12.88 (0.26)) than Adaptive/Resilient Phenotype (13.80 (0.30)) *B* = -0.91, 95% CI -1.66 to -0.16, SE = 0.38, *t* = -2.40, *p*=.017, Cohen's f^2 = 0.02
Emotional Exhaustion (EE)	1	274.95	149.66	*<.001*	Emotional exhaustion scores were higher in the At-Risk (11.97 (0.30)) than Adaptive/Resilient Phenotype (6.54 (0.33)) *B* = 5.43, 95% CI 4.56 to 6.30, SE = 0.44, *t* = 12.23, *p*<.001, Cohen's f^2 = 0.55
Depersonalization (DEP)	1	266.64	128.36	*<.001*	Depersonalization scores were higher in the At-Risk (8.00 (0.30)) than Adaptive/Resilient Phenotype (2.95 (0.34)) *B* = 5.05, 95% CI 4.17 to 5.93, SE = 0.45, *t* = 11.33, *p*<.001, Cohen's f^2 = 0.45
**Depressive symptoms**[3]	1	269.97	156.19	*<.001*	Depressive symptom scores were higher in the At-Risk (7.27 (0.28)) than Adaptive/Resilient Phenotype (1.94 (0.33)) *B* = 5.33, 95% CI 4.49 to 6.18, SE = 0.43, *t* = 12.50, *p*<.001, Cohen's f^2 = 0.55
**Anxiety symptoms**[4]	1	313.98	150.26	*<.001*	Anxious symptom scores were higher in the At-Risk (7.42 (0.30)) than Adaptive/Resilient Phenotype (1.94 (0.34)) *B* = 5.48, 95% CI 4.60 to 6.36, SE = 0.45, *t* = 12.26, *p*<.001, Cohen's f^2 = 0.54
**PTSD symptoms**[5]	1	277.33	126.71	*<.001*	PTSD symptom scores were higher in the At-Risk (21.54 (0.87)) than Adaptive/Resilient Phenotype (6.92 (1.00)) *B* = 14.62, 95% CI 12.06 to 17.17, SE = 1.30, *t* = 11.26, *p*<.001, Cohen's f^2 = 0.46

Test statistics are presented as mean (SD). [1]BRCS total scores: Brief Resilience Coping Scale, range 4-20; [2]MBI-9 domain scores: Maslach Burnout Inventory, range 0-18; [3]PHQ-8 total scores: Patient Health Questionnaire, total range 0-24; [4]GAD-7 total scores: General Anxiety Disorder-7, total range 0-21; [5]PCL5 total and subcluster scores: PTSD Checklist for DSM5, total range 0-80. Cohen's f^2 effect sizes were calculated following guidelines of GroB & Möller [5].

S6. Performance metrics for each classification machine learning model during cross-validation on the test set.

Classifier Model	Accuracy	F1	Precision	Recall
SVC	0.60 (0.11)	0.75 (0.08)	0.68 (0.07)	0.87 (0.13)
Logistic Regression Elastic Net	0.55 (0.12)	0.55 (0.29)	0.57 (0.33)	0.54 (0.30)
Logistic Regression L1	0.60 (0.11)	0.71 (0.09)	0.72 (0.11)	0.72 (0.10)
XGBoost	0.58 (0.07)	0.73 (0.06)	0.68 (0.05)	0.80 (0.12)
VotingClassifier	0.83 (0.06)	0.87 (0.05)	0.87 (0.07)	0.87 (0.03)

Cross-validated performance of all tested classifier models including SVM (SVC), Logistic Regression with L1 and Elastic-net regularization, XGBoost, and the VotingClassifier on the test set.

S7. Sensitivity analyses: the potential impact of demographic and clinical factors on the study results.

To evaluate potential impact of demographic and clinical factors on our results, we performed additional sensitivity analyses comparing the analytic subsamples with and without available video data (S7.1), and to examine whether potential demographic bias may have influenced model outputs (S7.2).

S7.1 Demographic and mental health measures across participants without available video data (Latent Profile Analysis-only) and those with available video data (full analytic sample).

	**Without video data**	**With video data**	
	LPA-only	*Full analytic sample*	**Statistics** (two-tailed)
**Participants**	**109**	**133**	
**Age** (years)	41.8 (12.0)	33.5 (8.0)[7]	*t*(182.49)=6.15, *p*<.001*
**Sex at birth**			
Female	79 (72.5%)	81 (60.9%)	*X*(1)=3.58, *p*=.058
Male	30 (27.5%)	52 (39.1%	
**Race**			
White	45 (41.3%)	60 (45.1%)	*p*=.533
Black or African American	18 (16.5%)	22 (16.5%)	
Asian	23 (21.1%)	33 (24.8%)	
Middle Eastern or North African	2 (1.8%)	1 (0.8%)	
American Indian/Native American	1 (0.9%)	1 (0.8%)	
Multiracial	4 (3.7%)	7 (5.3%)	
Other	7 (6.4%)	2 (1.5%)	
Unknown	3 (2.8%)	1 (0.8%)	
Declined to respond	6 (5.5%)	6 (4.5%)	
**Ethnicity**			
Hispanic or Latino	24 (22.0%)	17 (12.8%)	*p*=.091
Non-Hispanic or -Latino	84 (77.1%)	111 (83.5%)	

Declined to respond	1 (0.9%)	5 (3.8%)	
**Education level**			
High school diploma/GED	2 (1.8%)	0	*p*=.113
Trade school/Vocational school	1 (0.9%)	0	
(Some) College	12 (11.0%)	6 (4.5%)	
College graduate	31 (28.4%)	43 (32.3%)	
Graduate school/professional school	62 (56.9%)	83 (62.4%)	
Declined to respond	1 (0.9%)	1 (0.8%)	
**Current working position**[1]			
Faculty physician	22 (20.2%)	16 (12.0%)	*p*<.001*
Resident physician	5 (4.6%)	44 (33.1%)	
Physician assistant	6 (5.5%)	6 (4.5%)	
Nurse practitioner	5 (4.6%)	2 (1.5%)	
Licensed registered nurse	32 (29.4%)	48 (36.1%)	
Social worker	1 (0.9%)	0	
Other	38 (34.9%)	17 (12.8%)	
**Years in current position**[1]	8.0 (7.6)[8]	4.5 (5.2)[9]	*t*(174.69)=4.13, *p*<.001*
**Assessments**	110	168	
MENTAL HEALTH STATUS			
**Resilience**[2]	14.5 (2.5)	15.3 (2.2)	F(1,250.47)=5.91, *p*=.016*
**Burnout**[3]			
Personal Accomplishment	13.4 (3.2)	13.4 (3.2)	F(1,223.60)=0.24, *p*=.628
Emotional Exhaustion	9.5 (4.5)	9.9 (4.6)	F(1,245.81)=0.20, *p*=.653
Depersonalization	5.2 (4.3)	6.4 (4.5)	F(1,203.45)=2.44, *p*=.120
**Depressive symptoms**[4]	4.3 (4.1)	5.6 (4.6)	F(1,274.02)=4.28, *p*=.040*
**Anxiety symptoms**[5]	4.7 (4.4)	5.4 (4.7)	F(1,210.25)=1.01, *p*=.316
**PTSD symptoms**[6]	14.8 (13.3)	15.9 (13.0)	F(1,277.66)=0.38, *p*=.539

Scores are displayed as mean (SD) for continuous variables or n(%) for categorical variables. Sex at birth was based on self-reports. [1]Measured during baseline assessment; [2]Measured with total scores on the Brief Resilience Coping Scale (BRCS, range 4-20); [3]Measured with domain scores on the Maslach Burnout Inventory (MBI-9, range 0-18); [4]Measured with total scores on the Patient Health Questionnaire (PHQ-8, range 0-24); [5]Measured with total scores on the General Anxiety Disorder (GAD-7, range 0-21); [6]Measured with total and subdomain scores on the PTSD Checklist for DSM5 (PCL5, range 0-80); [7]Information for n=2 participants missing; [8]Information for n=5 participants missing; [9]Information for n=1 participant missing. p=0.05.

To evaluate potential differences between analytic subgroups due to video data availability we compared participants with available video data who were included in both the LPA and temporal features machine learning analyses (full analytic sample) with participants without available video data who were included in the LPA but not in the machine learning analyses (LPA-only sample). Group differences were examined using Linear Mixed Models with First-Order Autoregressive

covariance structure for continuous mental health measures, Independent Samples T-test for continuous demographic measures (age and years working in the current position), and Pearson's Chi-square or Fisher-Freeman-Halton Exact tests for categorical demographic variables (sex at birth, race, ethnicity, educational level, current working position).

Participants in the full analytic sample were younger than those in the LPA-only sample (mean age (SD) = 33.5 (8.0) vs. 41.8 (12.0), $t(182.49)=6.15$, $p<.001$) and had worked fewer years in their current position (mean years (SD) = 4.5 (5.2) vs. 8.0 (7.6), $t(174.69)=4.13$, $p<.001$). They were also more frequently resident physicians (33.1% vs. 4.6%), $p<.001$) and less frequently categorized in other positions (12.8% vs. 34.9%), $p<.001$). In addition, participants in the full analytic sample reported slightly higher resilience scores (estimated marginal means [SE] = 15.20 [0.19] vs. 14.49 [0.22], B = 0.71, 95% CI [0.13, 1.28], SE = 0.29, t = 2.43, p=.016) and depressive symptom scores (estimated marginal means [SE] = 5.47 [0.36] vs. 4.37 [0.41], B = 1.10, 95% CI [0.05, 2.14], SE = 0.53, t = 2.07, p=.040). However, despite reaching statistical significance, there was no clinically significant difference in resilience and depressive symptom scores between the groups. Effect sizes were small (Cohen's f^2 = 0.02 for both comparisons, calculated following guidelines of GroB & Möller [5]), and mean differences amounted to approximately one point on each scale. Additionally, both groups remained within the same clinical range (medium resilient coping and minimal-to-mild depression), further indicating that these differences are unlikely to be clinically important. Similarly, the differences observed in specific occupational categories primarily reflect variation in the proportion of resident physicians rather than a substantial shift in the overall composition of our healthcare worker population across analytical groups.

To evaluate the potential impact of these differences on the machine learning model performance, we conducted an additional sensitivity analysis in the full analytic sample using a Linear Mixed Model with first-order autoregressive covariance structure. We categorized age into four groups (18-25, 26-34, 35-49, and 50+ years) to reflect early-, mid-, and later-career stages. Prediction probabilities of the best-performing VotingClassfier model did not significantly differ across age groups ($F(3,131.62)=1.20$, p=.314), suggesting that age- and career-stage-related differences were unlikely to explain the machine learning findings.

S7.2 Evaluation of demographic subgroup effects on model prediction probabilities.

To examine whether potential demographic bias may have influenced model outputs in the present study, we conducted an additional sensitivity analysis to examine whether machine learning prediction probabilities derived from the facial expression features differed across racial groups. Our full analytic sample included participants from diverse racial background (White (45.1%), Black or Africa American (16.5%), Asian (24.8%), Middle Eastern or North African (0.8%), American

Indian/Native American (0.8%), Multiracial (5.3%), and Other (1.5%), Unknown (0.8%), or declined to respond (4.5%). We categorized race in four groups (White 45.1%, Black or African American 16.5%, Asian 24.8%, and Other [combined remaining categories], 13.7%) and used a Linear Mixed Model with first-order autoregressive covariance structure to evaluate potential race-related influences on model predictions.

Prediction probabilities of the best-performing VotingClassfier model did not differ significantly across racial groups ($F(3,123.73)=0.96$, $p=.412$), suggesting that the model produces similar prediction probabilities and provides reassurance that model outputs were broadly comparable across the racial groups represented in our sample.

References

1. Nylund KL, Asparouhov T, Muthén BO. Deciding on the number of classes in latent class analysis and growth mixture modeling: A monte carlo simulation study. Struct Equ Modeling. 2007;14(4):535–569. https://doi.org/10.1080/10705510701575396
2. Celeux G, Soromenho G. An entropy criterion for assessing the number of clusters in a mixture model. J Classif. 1996;13(2):195–212. https://doi.org 10.1007/BF01246098
3. Asparouhov T, Muthén B. Auxiliary variables in mixture modeling: Three-step approaches using Mplus. Struct Equ Modeling. 2014;21(3):329–341. https://doi.org/10.1080/10705511.2014.915181
4. Yang C-C. Evaluating latent class analysis models in qualitative phenotype identification. Comput Stat Data Anal. 2006;50(4):1090–1104. https://doi.org/10.1016/j.csda.2004.11.004
5. Groß J, Möller A. Effect size estimation in linear mixed models. METRON. 2025;83:353–363. https://doi.org/10.1007/s40300-025-00295-w
6. Cheong J, Jolly E, Xie T, Byrne S, Kenney M, Chang L. Py-Feat: Python Facial expression analysis toolbox. Affect Sci. 2023;4(4):781–796. https://doi.org/10.1007/s42761-023-00191-4
7. Chen I, Chen Y, Liao S, Lin Y. Development of digital biomarkers of mental illness via mobile apps for personalized treatment and diagnosis. J Pers Med. 2022;12(6):936:936. https://doi.org/10.3390/jpm12060936
8. Baltrusaitis T, Zadeh A, Chong Lim Y, Morency L-P. OpenFace 2.0: Facial behavior analysis toolkit. 2018 13th IEEE International Conference on Automatic Face & Gesture Recognition (FG 2018), Xi'an, China. 2018:59–66. https://doi.org/10.1109/FG.2018.00019
9. Gajarsky T. Facetorch: A python library for analyzing faces using PyTorch. GitHub - GitHub Repository. 2024.
